# Ring artifacts removal in X-ray-induced acoustic computed tomography

**DOI:** 10.1142/s1793545822500171

**Published:** 2022-03-30

**Authors:** Prabodh Kumar Pandey, Hari Om Aggrawal, Siqi Wang, Kaitlyn Kim, An Liu, Liangzhong Xiang

**Affiliations:** * Department of Radiological Sciences, University of California, Irvine, CA 92697, USA; † Institute of Mathematics and Image Computing, University of Lübeck, Germany; ‡ Independent Technical Consultant, India; § Department of Biomedical Engineering, University of California, Irvine, CA 92617, USA; ¶ Department of Radiation Oncology, City of Hope National Medical Center, Duarte CA 91010, USA; || Beckman Laser Institute, University of California, Irvine, CA 92612, USA

**Keywords:** X-ray-induced acoustic computed tomography (XACT), ring artifacts, artifacts correction

## Abstract

X-ray-induced acoustic computed tomography (XACT) is a hybrid imaging modality for detecting X-ray absorption distribution via ultrasound emission. It facilitates imaging from a single projection X-ray illumination, thus reducing the radiation exposure and improving imaging speed. Nonuniform detector response caused by the interference between multichannel data acquisition for ring array transducers and amplifier systems yields ring artifacts in the reconstructed XACT images, which compromises the image quality. We propose model-based algorithms for ring artifacts corrected XACT imaging and demonstrate their efficacy on numerical and experimental measurements. The corrected reconstructions indicate significantly reduced ring artifacts as compared to their conventional counterparts.

## 1. Introduction

X-rays have been vital for biomedical research by facilitating imaging through computerized tomography (CT) scans as well as treating tumors through radiation therapy. The carcinogenic property of X-rays motivates the need for imaging modalities which requires low radiation dose. X-ray-induced acoustic tomography (XACT) is one such imaging technique based on X-ray excitation and ultrasonic detection. Medical ultrasound images are known to have a good spatial resolution but inherently carry features of poor contrast. XACT combines high X-ray imaging contrast with high ultrasonic spatial resolution. When absorbed, temporally short X-ray pulses generate ultrasound (US), which can be sensed via ultrasonic transducers.^1^ Acoustic detection enables XACT to achieve three-dimensional imaging from a single X-ray projection, thus reducing the radiation exposure and the experimental complexity associated with rotating X-ray source(s) and detectors as needed by CT.^2^

Since its first demonstration,^1^ XACT has found applications in tomographic imaging^3–10^ as well as radiation dosimetry.^11–17^ The aim of XACT reconstruction is to obtain the map of the initial pressure source (which is proportional to X-ray energy deposition (XED)) from the pressure signals acquired at multiple spatial locations of the detector grid. Traditionally, this is achieved using the back-projection (BP)^1^ and time-reversal (TR)^18^ algorithms. TR algorithm is numerically implemented by simulating time-reversed propagation of the acoustic waves, which is slow and computationally demanding. Therefore, although not as accurate as TR, BP is the commonly used reconstruction algorithm. An efficient, GPU accelerated BP-based XACT reconstruction was recently reported by Lee *et al.*^6^ Model-based algorithms (model back-projection (MBP) and matrix-free least squares minimization (MF-LSQR) algorithms)^9^ have also been proposed to solve the XACT reconstruction problem. Such schemes can efficiently incorporate finite detector aperture and acoustic inhomogeneities as well as regularization priors to ameliorate the noisy and limited view artifacts.^9^ The contribution of this work is developing a weighted MF-LSQR algorithm that inhibits the ring artifacts in the XACT reconstruction which are caused due to the multichannel interference of the detection system.

In 2013, the first report of XACT's capability for imaging was presented by Xiang *et al.*^1^ They irradiated a chicken breast tissue (with lead targets concealed in it) with pulsed X-rays emitted from a medical linear accelerator and collected XA signals by scanning a single transducer element around a circle. Back-projected reconstructions revealed the positions and sizes of the concealed targets. Since then, several studies have been performed to study the feasibility of XACT-based biomedical imaging such as imaging microcalcifications in human breast to diagnose breast cancer,^5^ high resolution imaging of tumor^3^ tagged with gold fiducial marker,^19^ and 3D bone imaging.^8^ XACT imaging was demonstrated using synchrotron as the X-ray source (pulse width of 30 ps with 2 ns repetition period) by Choi *et al.* which manages to overcome the shortcomings of the commonly used XACT sources: the medical linear accelerators (LINACs) and portable X-ray tubes.^20^

Other than the tomographic imaging, another potential application of XACT is radiation dosimetry which is motivated by the proportionality of the XA signal strength with X-ray radiation dose.^1^ This makes XACT desirable for monitoring and planning radiotherapy. Several numerical studies have demonstrated the feasibility of XACT assisted radiation dosimetry.^11,18,21–25^ The first XA signal due to absorption of a single X-ray pulse from a Linac in water was demonstrated^12^ and the XED in water was reconstructed using BP. Systematic characterization of such a setup for *in vivo* radiation dosimetry was performed by Hickling *et al.*^13^ Application of XACT for imaging (relative) radiation dose map in a biological tissue was first realized by Lei *et al.*^15^ In 2017, an XACT dosimeter was developed by Kim *et al.*^14^ which employed a clinical Linac as the excitation source and a spherically focused transducer was scanned to collect the XA signals; such a device was later patented by the group.^16^ These results indicate the immense potential of XACT for *in vivo* dosimetry. Moreover, the clinical Linacs have been shown to generate strong XA signals and hence the clinical radiotherapy setups only need to integrate the coupling medium and a transducer array (detection grid) for clinical translation of XACT.

A majority of XACT setups employ mechanical scanning of a single transducer to obtain pressure measurements at various spatial locations.^1,14,16^ This, however, is tedious and time consuming. To obtain faster imaging speed, it is imperative to obtain the pressure measurements at multiple locations of the detector grid simultaneously. It can be achieved using multichannel data acquisition systems (DAQs). However, the nonuniform response due to the interference between electronics associated with multichannel DAQ and amplifier systems may cause all sensor elements to have a simultaneous instantaneous gain in the acquired signal. This results in vertical lines (stripe pattern) in the sinogram and artifacts in the reconstructed image. Particularly, in circular detection geometry, this causes ring artifacts in the images. Ring array of transducers allows 360° capturing of the acoustic waves originating from the same plane as the ring, thus facilitating 2D reconstruction of the pressure source map in this plane. Such a geometry is commonly used for small animal imaging^26,27^ as well as for imaging human finger.^28,29^ Ring artifacts have also been reported in microwave-induced thermoacoustic tomography (TAT).^30^ In another sister imaging modality photoacoustic tomography (PAT), which shares similar physics as XACT and TAT, ring artifacts have been reported.^31,32^ However, as compared to XACT, the ring artifacts in PAT images are less likely because of relatively better SNRs in the acoustic signals due to shorter excitation pulse widths, better contrast agents (intrinsic and extrinsic), higher excitation pulse energy. The multichannel interference of the detection electronics can be suppressed using better shielded cables, proper cable routing, and proper grounding of the sensors. However, even with improved hardware, the ring artifacts may affect the image quality of low contrast targets and hence, an algorithm that corrects these artifacts in the XACT images is desirable.

Since CT algorithm shares generic similarity with XACT (and TAT), nonuniformity in CT measurements also produce ring artifacts in the images. Nonuniformity in CT is caused due to crosstalk and reverberation effects^33^ or manufacturing issues such as faulty or miscalibrated detector elements.^34^ There have been many reports on ring artifact correction methods in CT imaging, some of them can be extended to the ring array-based XACT imaging. Ring artifact correction can be implemented either on the sinogram domain^34–37^ or on the reconstructed image domain.^38–40^ Interestingly, such studies in the context of XACT are rather limited. A sinogram-based algorithm that requires additional signal processing was proposed by Eldib *et al.*^41^ Additional signal processing as well intermediate reconstructions associated with this algorithm results in increased computation time. Moreover, this scheme was found to be inefficient for the testcases mimicking strong electromagnetic interference and hence severe ring artifacts. In PAT, Tzoumas *et al.* demonstrated an algorithm that implements a three-stage correction of the PA signals for denoising which reduces the ring artifacts in the reconstructions.^31^ Another deep learning-based algorithm was proposed by Dehner *et al.* for ring artifacts correction in PAT.^32^ However, both these algorithms increase the image reconstruction time and the deep learning algorithm in particular, needs a large amount of experimental training datasets which is computationally expensive.

Therefore, aiming for fast and efficient XACT imaging, a correction method combined in the reconstruction algorithm itself is more desirable. The contribution of this work is the development of model-based weighted least squares (LS) minimization approach to produce ring artifacts corrected reconstructions from corrupt, noisy XA measurements. The efficacy of the proposed algorithm is demonstrated on numerical as well as experimental XA datasets.

The rest of this paper is structured as follows. Section 2 deals with the problem formulation and derivation of the ring artifacts corrected reconstruction algorithm. The numerical and phantom XACT experimental studies are discussed in Secs. 3 and 4, respectively. Concluding remarks are offered in Sec. 5.

## 2. Mathematical Formulation

### 2.1. Forward problem

Assuming instantaneous heating, the time domain acoustic equation is written as^42^

(1) $$\frac{\partial^{2}p\text{(}\vec{r}\text{,}\;t\text{)}}{\partial t^{2}}-v^{2}\nabla^{2}p\text{(}\vec{r}\text{,}\;t\text{)}=\Gamma H\text{(}\vec{r}\text{)}\frac{\partial \delta \text{(}t\text{)}}{\partial t}\text{,}$$

where $\text{Γ(}=v^{2}\beta \text{/}C_{p}\text{)}$ is the Gruneisen parameter, v is the sound speed, β is the volumetric expansion coefficient, $C_{p}$ is the specific heat at constant pressure and H is the XED. The solution to Eq. (1) is given by^43^

(2) $$p\text{(}\vec{r}\text{,}\;t\text{)}=\frac{\Gamma }{4\pi v}\frac{\partial }{\partial t}\left(\frac{1}{vt}\int_{S\text{(}\vec{r}\text{,}\;t\text{)}}H\left({\vec{r}}^{\prime }\right)dS^{\prime }\text{(}t\text{)}\right);\left|\vec{r}-{\vec{r}}^{\prime }\right|=vt.$$

where $S^{\prime }\left(t\right)$ denotes a time-dependent spherical surface centered at a detector (located at $\vec{r}$) such that $\left|\vec{r}-{\vec{r}}^{\prime }\right|=vt$. If the pressure source lies in a plane, Eq. (2) reduces to its 2D analog where the integral in Eq. (2) represents the integration of the pressure distribution in the domain of interest on a circular arc of radius vt. Setting $\frac{\Gamma }{4\pi v}$ as unity, and further solving Eq. (2) can be written as

(3) $$p\text{(}\vec{r}\text{,}\;t\text{)}=\frac{\partial }{\partial t}\left(\int_{S(\vec{r}\text{,}t\text{)}}H\left({\vec{r}}^{\prime }\right)d\theta \right);\;\left|\vec{r}-{\vec{r}}^{\prime }\right|=vt.$$

Equation (3) can be simplified in the discrete domain^9^ to obtain the acoustic measurements ${\underline{p}}^{\text{(}q\text{)}}$ (size: $N_{d}\times 1$; $N_{d}$ being the number of detectors) at qth time-step ($t^{\text{(}q\text{)}}=q\text{Δ}t$, Δt being the sampling period) due to the discrete XED map $\underline{h}$ (size: N×1; N being the number of nodes) as

(4) $${\underline{p}}^{\text{(}q\text{)}}={𝓜}^{\text{(}q\text{)}}\underline{h}\text{,}$$

where ${𝓜}^{\text{(}q\text{)}}$ is the measurement operator (size: $N_{d}\times N$) relating the pressure signal at all the detectors.

### 2.2. Origin of ring artifacts

**Fig. 1.**
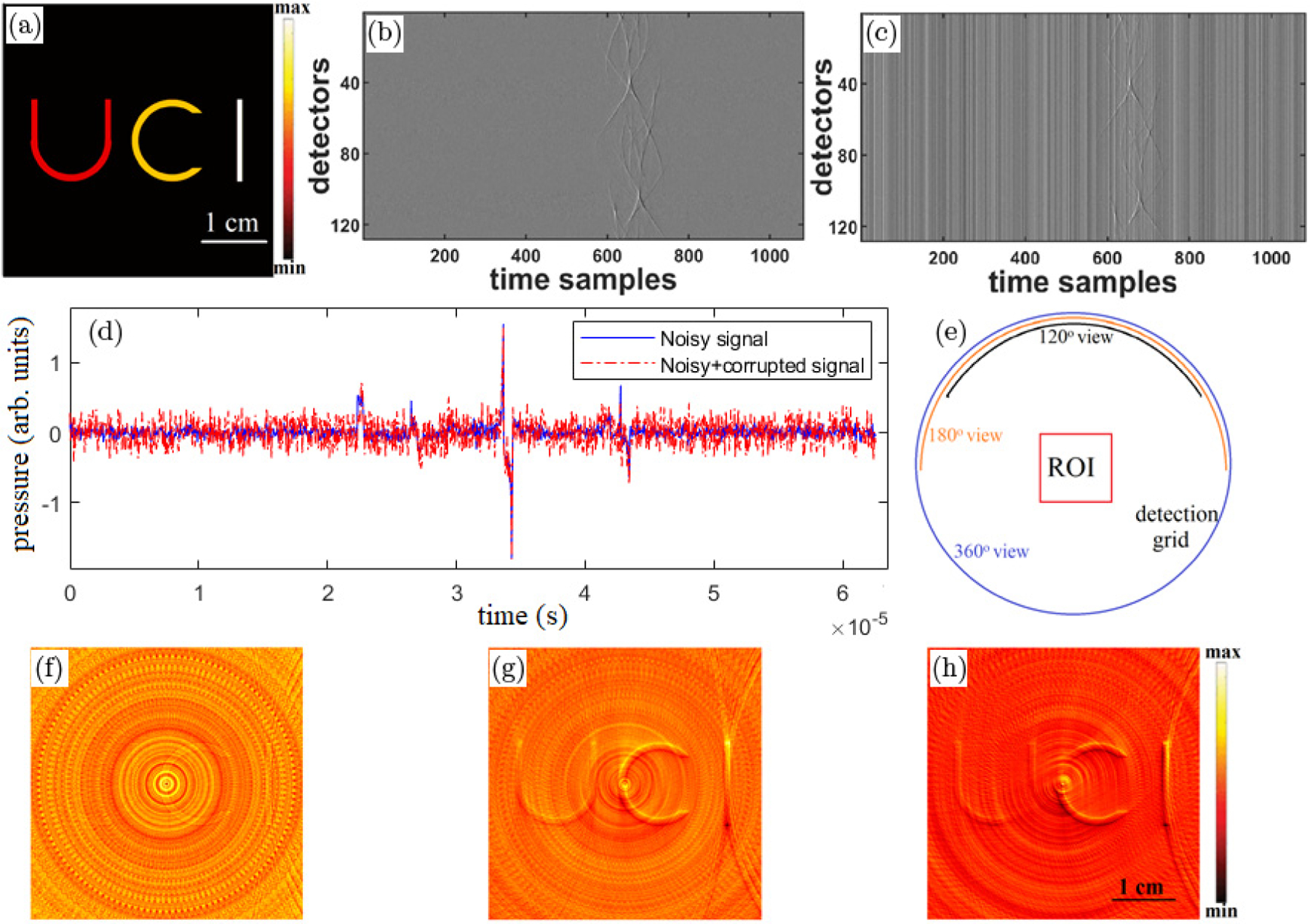
(a) True pressure source, sinograms of the (b) noisy and (c) corrupted XA measurement data (SNR = 5 dB), (d) noisy and corrupted XA signals from a detector, (e) full and partial view geometries and BP reconstructions from (f) 360°, (g) 180°, and (h) 120° view measurements.

In XACT, the measurements have a systematic error in the form of an offset due to the time-varying response of detectors. This offset appears as vertical lines in a sinogram; see Fig. 1(c) and it leads to ring artifacts in the reconstruction for the circular geometry of detectors (Figs. 1(e)–1(g)).

We assume that each detector has the same time-varying detector response which introduces an offset $z^{\text{(}q\text{)}}\in \mathbb{R}$ at all detectors. With this assumption, we say that the measurements ${\underline{p}}^{\text{(}q\text{)}}$ holds

(5) $${\underline{p}}^{\text{(}q\text{)}}={𝓜}^{\text{(}q\text{)}}\underline{h}+z^{\text{(}q\text{)}}𝟙+e.$$

We assume that the measurements are corrupted by additive and Gaussian distributed noise vector $e\sim 𝓝\left(0\text{,}\;\sigma^{2}I\right)$ and $z^{\left(q\right)}$ is a zero-mean Gaussian distributed random variable; $z^{\text{(}q\text{)}}∼𝓝\left(0\text{,}\;\beta^{2}\right)$. Each measurement conditioned on the same random variable $z^{\left(q\right)}$, hence we have $z^{\left(q\right)}𝟙∼𝓝\left(0,\beta^{2}𝟙{𝟙}^{⊤}\right)$. 𝟙 denotes a $N_{d}$-dimensional vector of ones and $I\in {\mathbb{R}}^{N_{d}\times N_{d}}$ an identity matrix where $N_{d}$ is the number of detectors.

Assuming the offset and measurement noise are independent random variables, ${\underline{p}}^{\text{(}q\text{)}}$ is also a Gaussian random variable with

(6) $${\underline{p}}^{\text{(}q\text{)}}∼𝓝\left({𝓜}^{\text{(}q\text{)}}\underline{h}\text{,}\;{\text{Σ}}^{\text{(}q\text{)}}\right);\;{\text{Σ}}^{\text{(}q\text{)}}=\beta^{2}𝟙{𝟙}^{⊤}+\sigma^{2}I.$$

Further assuming that the measurements at every time instants are independent, the joint distribution of $\underline{p}$ is also normally distributed with mean $M\underline{h}$ and a block-diagonal covariance matrix

(7) $$\text{Σ}=\text{blkdiag}\left({\text{Σ}}^{\text{(}q=1\text{)}}\text{,}\ldots \text{,}\;{\text{Σ}}^{\left(q=N_{t}\right)}\right)\text{,}$$

with $N_{t}$ being the total number of time samples in the measured signals.

### 2.3. Reconstruction algorithm

Given the joint distribution of $\underline{p}$ given $\underline{h}$, the maximum likelihood estimate of $\underline{h}$ is given by

(8) $$\underline{\hat{h}}=\underset{\underline{h}}{\text{arg}\;\text{min}}∥{\text{Σ}}^{-1}\text{(}M\underline{h}-\underline{p}\text{)}{∥}_{2}^{2}.$$

We use the iterative solver LSQR to minimize (8) by solving the linear system

(9) $$WM\underline{h}=W\underline{p}\text{,}$$

where the weight matrix $W={\text{Σ}}^{-1}=\text{blkdiag}\left(S^{\left(1\right)}\text{,}\ldots \text{,}\;S^{\left(N_{t}\right)}\right)$ follows from the Sherman–Morrison formula^44^ that yields

(10) $$S^{\text{(}q\text{)}}=\sigma^{-2}\left(I-\frac{1}{\sigma^{2}\text{/}\beta^{2}+N_{d}}𝟙{𝟙}^{⊤}\right).$$

β→0 indicates minimal offset resulting $S^{\text{(}q\text{)}}\to I$; the effect of offset is completely neglected in the joint distribution of $\underline{p}$. The stripe pattern in the sinogram, which is the indicator of the offsets, is prominent only if σ≤β, i.e., the offsets are dominant over the noise in the measurements. In this case, and given the number of detectors $N_{d}\gg 1$, the weight matrix reduces to

(11) $$S\;\text{:=}\;S^{\text{(}q\text{)}}=\sigma^{-2}\left(I-\frac{1}{N_{d}}𝟙{𝟙}^{⊤}\right)\;\forall q\in \left[1\text{,}\;N_{t}\right].$$

In an ideal case, if the residual has only offset, i.e., ${𝓜}^{\text{(}q\text{)}}\underline{h}-{\underline{p}}^{\text{(}q\text{)}}=c1$ where c∈ℝ, the minimizing function Eq. (8) results to $S^{\text{(}q\text{)}}\left({𝓜}^{\text{(}q\text{)}}\underline{h}-{\underline{p}}^{\text{(}q\text{)}}\right)=0$ for each qth time steps. Hence, with weight matrix Eq. (11), the minimizing function Eq. (8) filters out offsets in the residual and in results reduces the ring artifacts in the estimate $\underline{h}$. A similar algorithm has been used for ring artifacts correction in X-ray CT

$$\bar{\underline{\begin{array}{l} \underline{\begin{array}{l} \mathrm{Algorithm}\;1.\;\text{Model-based}\;\text{(MF-LSQR)}\;\text{ring}\;\text{arti-}\\ \text{facts}\;\text{corrected}\;\text{reconstruction}\;\text{from}\;\text{corrupted}\;\text{mea-}\\ \text{surement}\;\text{data}\;\underline{p.}\;\;\;\;\;\;\;\;\;\;\;\;\;\;\;\;\; \end{array}}\\ \;\;\text{1:}\;\mathrm{procedure}\;\text{COMPUTING}\;\underline{\hat{h}};\;\text{GIVEN}\;\text{THE }\\ \;\;\text{BOUNDARY XA DATA }\underline{p}\\ \;\;\text{2:}\;\;S\leftarrow I-\frac{1}{N_{d}}𝟙\;{𝟙}^{T}\;\text{(Preparing}\;\text{the}\;\text{weight}\\ \;\;\text{matrix}\;\text{using}\;\text{Eq}.\;\text{(11)) }\\ \;\;\text{3:}\;\;P\leftarrow reshape\left(\underline{p}\text{,}\;N_{d}\text{,}\;N_{t}\right)\\ \;\;\text{4:}\;\;B\leftarrow SP\\ \;\;\text{5:}\;\;\text{Solve: }\underline{\hat{h}}=\text{arg}\;{\text{min}}_{\underline{h}}∥B-\text{S}\text{reshape}\\ \;\;\left(M\underline{h}\text{,}\;N_{d}\text{,}\;N_{t}\right){∥}_{2}^{2}\;\text{using}\;\text{matrix-free}\;\text{LSQR}\\ \;\;\text{minimization}.^{9}\\ \;\;\text{6:}\;\;\mathrm{return}\;\underline{\hat{h}}\\ \;\;\text{7:}\;\;\mathrm{end}\;\mathrm{procedure}\; \end{array}}}$$

$$\bar{\underline{\begin{array}{l} \underline{\begin{array}{l} \mathrm{Algorithm}\;2.\;\text{MBP-based}\;\text{ring}\;\text{artifacts}\;\text{corrected}\\ \text{reconstruction}\;\text{from}\;\text{corrupted}\;\text{measurement}\;\text{data}\;\underline{p.}\;\;\;\; \end{array}}\\ \;\;\text{1:}\;\;\;\;\;\mathrm{procedure}\;\text{COMPUTING}\;\underline{\hat{h}};\;\text{GIVEN}\;\text{THE }\\ \;\;\;\;\;\;\text{BOUNDARY XA DATA }\underline{p}\\ \;\;\text{2:}\;\;S\leftarrow I-\frac{1}{N_{d}}𝟙\;{𝟙}^{T}\;\text{(Preparing}\;\text{the}\;\text{weight}\\ \;\;\;\;\;\;\text{matrix}\;\text{using}\;\text{Eq}.\;\text{(11)) }\\ \;\;\text{3:}\;\;P\leftarrow reshape\left(\underline{p}\text{,}\;N_{d}\text{,}\;N_{t}\right)\\ \;\;\text{4:}\;\;B\leftarrow SP\\ \;\;\text{5:}\;\;\underline{\hat{h}}\leftarrow M^{T}B\text{(:)}.\\ \;\;\text{6:}\;\;\mathrm{return}\;\underline{\hat{h}}\\ \;\;\text{7:}\;\;\;\;\mathrm{end}\;\mathrm{procedure}\; \end{array}}}$$

imaging.^45^ Eq. (9) is solved using matrix-free LSQR approach described by Pandey *et al.*^9^ and corresponding steps are provided in Algorithm 2 Matrix-free LSQR minimization employs on the fly computation of the matrix-vector products of the type $M\underline{u}$ and $M^{T}\underline{v}$. In order to ameliorate the noisy artifacts in the images, Laplacian regularizer was employed while evaluating MF-LSQR reconstructions. The algorithms for these computations have been thoroughly discussed in our recent publication.^9^

## 3. Numerical Studies

### 3.1. Ring artifacts correction

Numerical studies were performed to validate the proposed correction algorithms. Simulations were carried out on MATLAB R2020a software. The codes were custom written to perform all the numerical studies as well for carrying out the reconstructions from the experimental measurements. We consider a square of side length 2 cm as the region of interest (ROI). A circle of radius 5 cm, concentric with the ROI is considered as the detection array with 128 detection-points uniformly distributed on its circumference. The initial pressure source chosen for this study is shown in Fig. 1(a). To simulate the acoustic signals (mimicking experimental measurements), the ROI was discretized at h=30 µm mesh resolution and each arc of integration was divided into $N_{q}\approx 1000$ quadrature elements. For model-based reconstructions, grid resolution was chosen to be h=60 µm with $N_{q}\approx 600$ quadrature points for computing the arc integrals. The numerical acoustic signal at each detector was recorded at $F_{s}=20\;\text{MHz}$ sampling frequency, and white Gaussian noise (background) was added to obtain signals with 5 dB SNR (using the MATLAB function: “awgn” —additive white Gaussian noise); corresponding sinogram is shown in Fig. 1(b). The multichannel interference is modeled as $z^{\left(q\right)}$ (in Eq. (5)) which is a zero-mean Gaussian distributed random variable. This random variable is added to the XA signal corresponding to each of the transducers which results in as vertical lines in the sinogram (Fig. 1(c)). The noisy and corrupted signals from a particular channel are plotted in Fig. 1(d). The corrupted signal though looks like a more noisy version of the noisy signal, the nonuniform response affects all the detectors equally at all the time instances thus yielding vertical lines in the sinogram. The conventional BP reconstructions corresponding to full and partial view geometries (Fig. 1(e)) are demonstrated in Figs. 1(f)–1(h). In the testcase demonstrated here, the nonuniformity response from the detectors is responsible for the artifacts. So, while more detectors mean better view and hence better reconstruction of the structures in the ROI, it also means more artifacts in the ROI. From a closer visual inspection of the reconstructed images, one can ascertain that the structures in the “UCI” symbol are indeed better reconstructed in the full view image (Fig. 1(f)) while limited view artifacts are visible in Figs. 1(g) and 1(h). However, the strong ring artifacts from the full view geometry eclipse the reconstructed structures-especially the lower contrast target “U”.

**Fig. 2.**
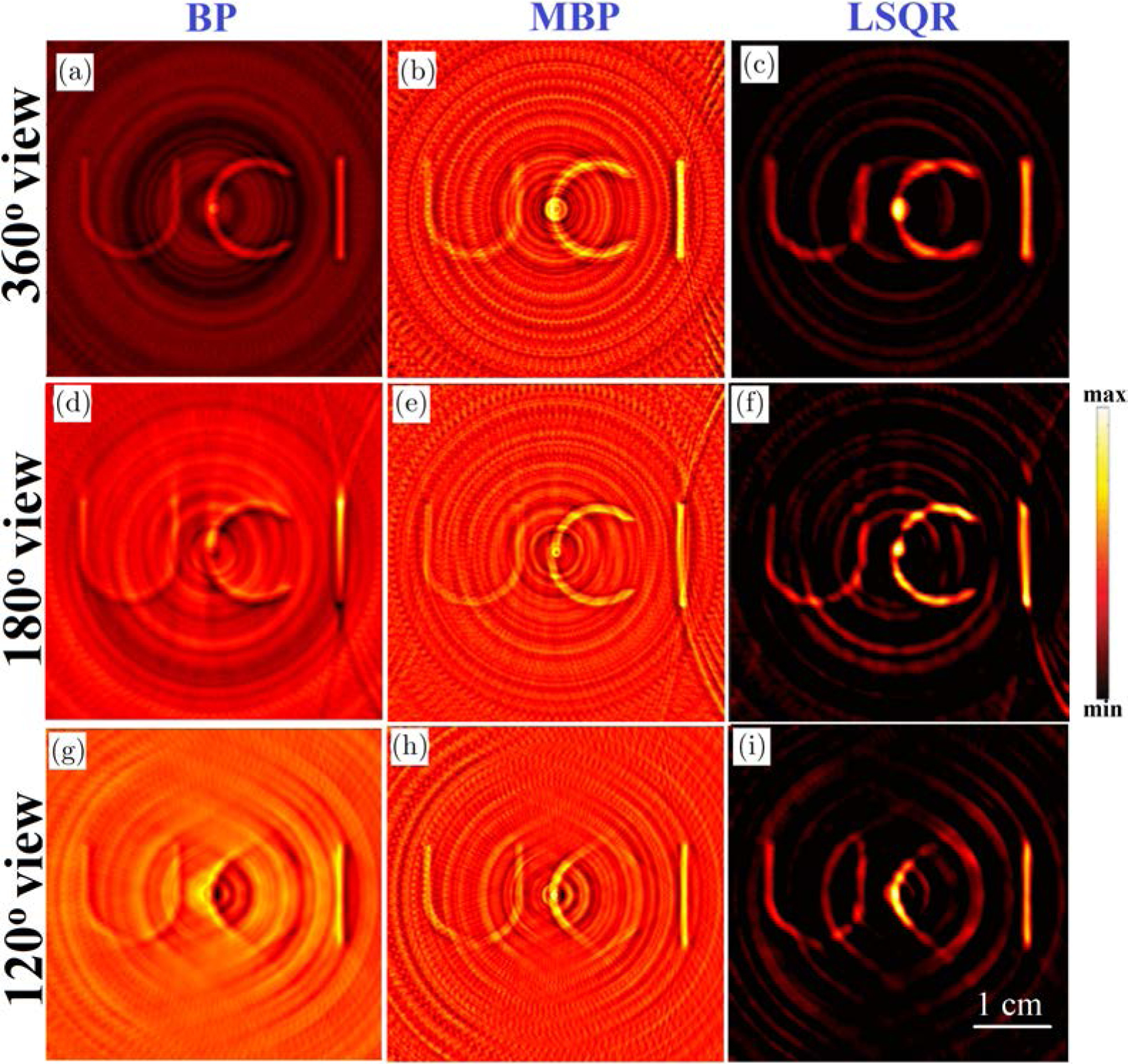
Numerical results from BW filtered measurements: Conventional (a), (d), (g) BP and (b), (e), (h) MBP and (c), (f), (i) MF-LSQR reconstructions for 360°, 180° and 120° view XA measurements.

Huang *et al.* stated that second-order Butterworth (BW) filtering of XA signals can reduce the ring artifacts in the reconstructions.^30^ This idea comes from the fact that the nonuniform response in the DAQ due to electromagnetic interference is typically included in the high frequency components of the XA signals. Therefore, BW filter-based smoothing of the XA signals will reduce the non-uniform response from the XA signals leading to reduced ring artifacts in the reconstructed images. Figure 2 shows the reconstructed cross-sections obtained from the XA data filtered using BW filter centered at 1 MHz frequency. As compared to the BP reconstruction from the raw, corrupted data, the BW filtered data yields reduced ring artifacts and better visibility of the structures in the reconstructions. However, the reconstructed images computed using the BP as well as the conventional model-based schemes, still carry significant ring artifacts which is undesirable. Moreover, the filtering also attenuates the frequency components of the XA signals originating from the true heat source well. This leads to the loss of quantitative information of the cross-sections.

**Fig. 3.**
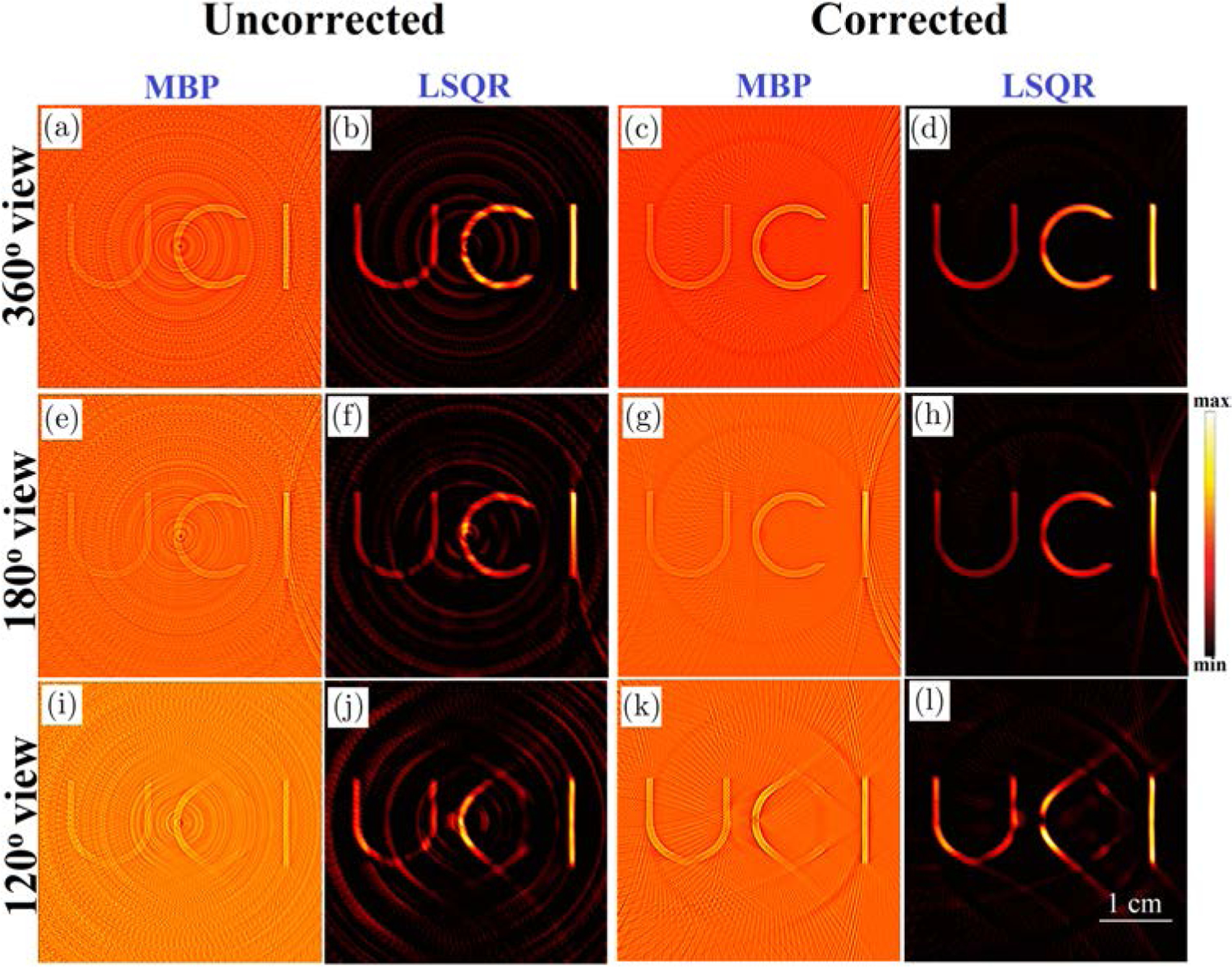
Numerical results: Conventional (a), (e), (i) MBP and (b), (f), (j) MF-LSQR reconstructions and ring-artifacts corrected (c), (g), (k) MBP and (d), (h), (l) MF-LSQR reconstructions for 360°, 180° and 120° view XA measurements.

The reconstructions obtained from the conventional and the proposed model-based algorithms (MBP and MF-LSQR algorithms) for full-and partial-view, raw (without BW filtering) XA measurements are demonstrated in Fig. 3. The conventional model-based reconstructions (Figs. 3(a), 3(b), 3(e), 3(f), 3(i) and 3(j)) display ring artifacts. However, as compared to the conventional BP reconstructions the smoothing effect of regularization suppresses the ring artifacts up to a certain extent.^9^ The efficacy of the proposed ring artifacts corrected model-based algorithms is evident in the MBP (Figs. 3(c), 3(g) and 3(k)) and LSQR reconstructions (Figs. 3(d), 3(h) and 3(l)) which display negligible ring artifacts. As discussed by Pandey *et al.*,^9^ the noniterative MBP reveals the structures in the ROI reasonably well, superior quantitative accuracy is achieved in the iterative LSQR reconstructions. It needs to be noted that the reconstructions obtained from the 120° view measurements display missing structures which are aligned normal to the detection grid. The cause of such artifacts is not the nonuniform detector response but the missing acoustic measurements.^9,46,47^ The correlation coefficients $\left(\rho \right)$^9^ of the uncorrected and corrected model-based reconstructions along with the model-based reconstructions obtained from the BW filtered data are tabulated in Table 1. Across all the test cases, the MF-LSQR being a quantitative reconstruction algorithm, yields higher ρ values as compared to the MBP counterparts. The BW filtering of XA signals attenuates the frequency components, thus leading to loss of quantitative information of the phantoms. This yields lower ρ values for BW filtered MF-LSQR reconstructions as compared to the uncorrected MF-LSQR ones. The corrected MF-LSQR reconstructions carry minimal ring artifacts and accurate quantitative information, thus yielding higher ρ values as compared to the BW filtered and uncorrected counterparts. For MBP algorithm, the BW filtered reconstructions show higher and comparable ρ values than their uncorrected and corrected counterparts. This can be attributed to the reduced noise in the XA measurements due to filtering. The ρ values across all the testcases decrease with the decreasing view due to the limited view artifacts in the reconstructed images. The LSQR reconstructions in this work were performed with Laplacian regularization, which favors smooth reconstructions and suppresses the noisy and streak artifacts. On the other hand, MBP reconstruction is a highly Tikhonov regularized solution. Unlike, Laplacian regularization, Tikhonov regularization simply seeks a low norm solution instead of a smoother solution. This is why as compared to the LSQR reconstructions, the MBP reconstructions have relatively more refined edges, but they also carry the streak and noisy artifacts.

### 3.2. Contrast and resolution of LSQR-based XACT reconstructions

**Fig. 4.**
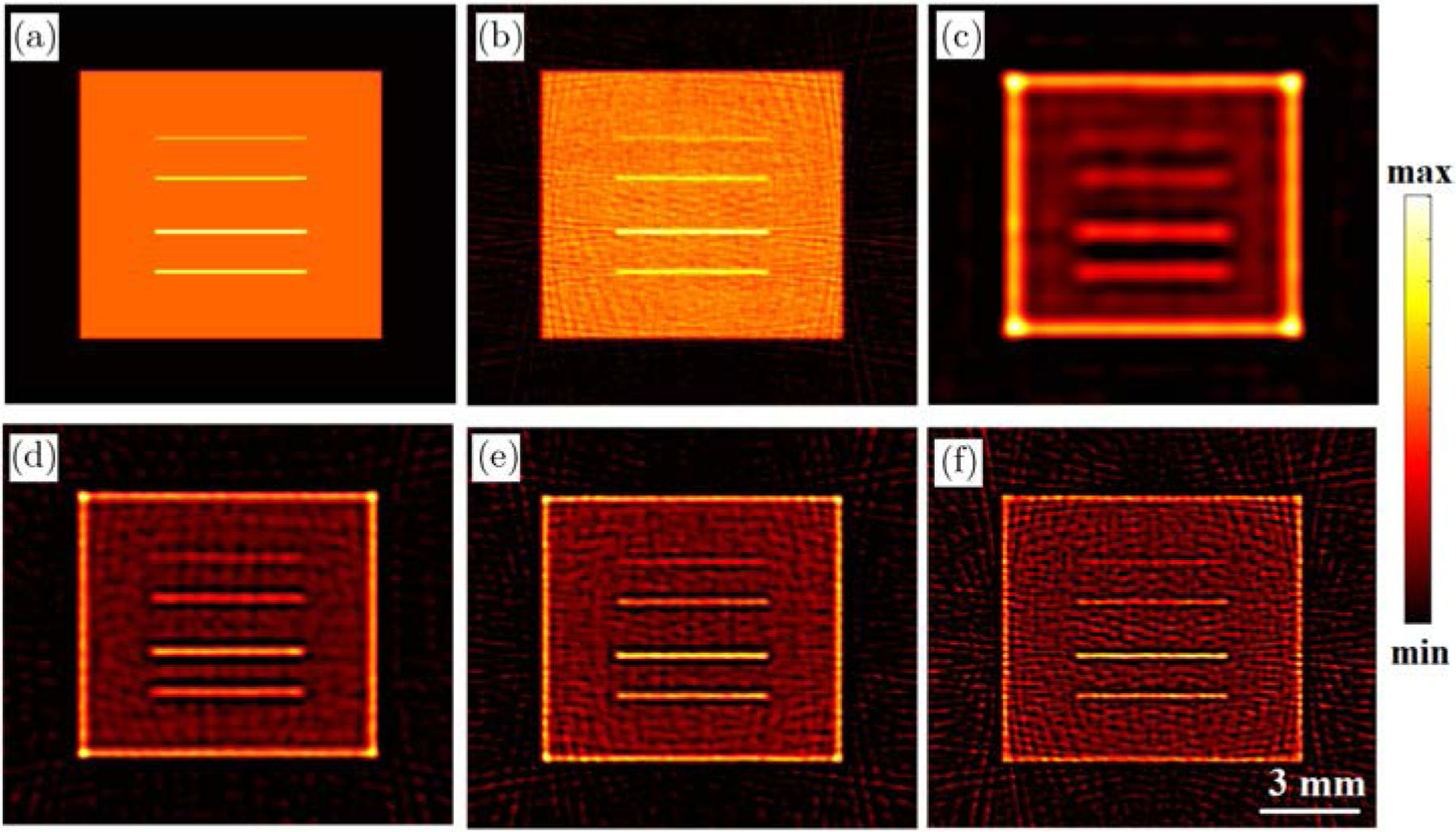
Numerical results: (a) True phantom, full-view LSQR reconstructions with numerical XACT data with (b) full frequency bandwidth, (c) $F_{c}=1\;\text{MHz}$ and 100% bandwidth, (d) $F_{c}=2\;\text{MHz}$ and 100% bandwidth, (e) $F_{c}=3\;\text{MHz}$ and 100% bandwidth and (f) $F_{c}=4\;\text{MHz}$ and 100% bandwidth.

**Fig. 5.**
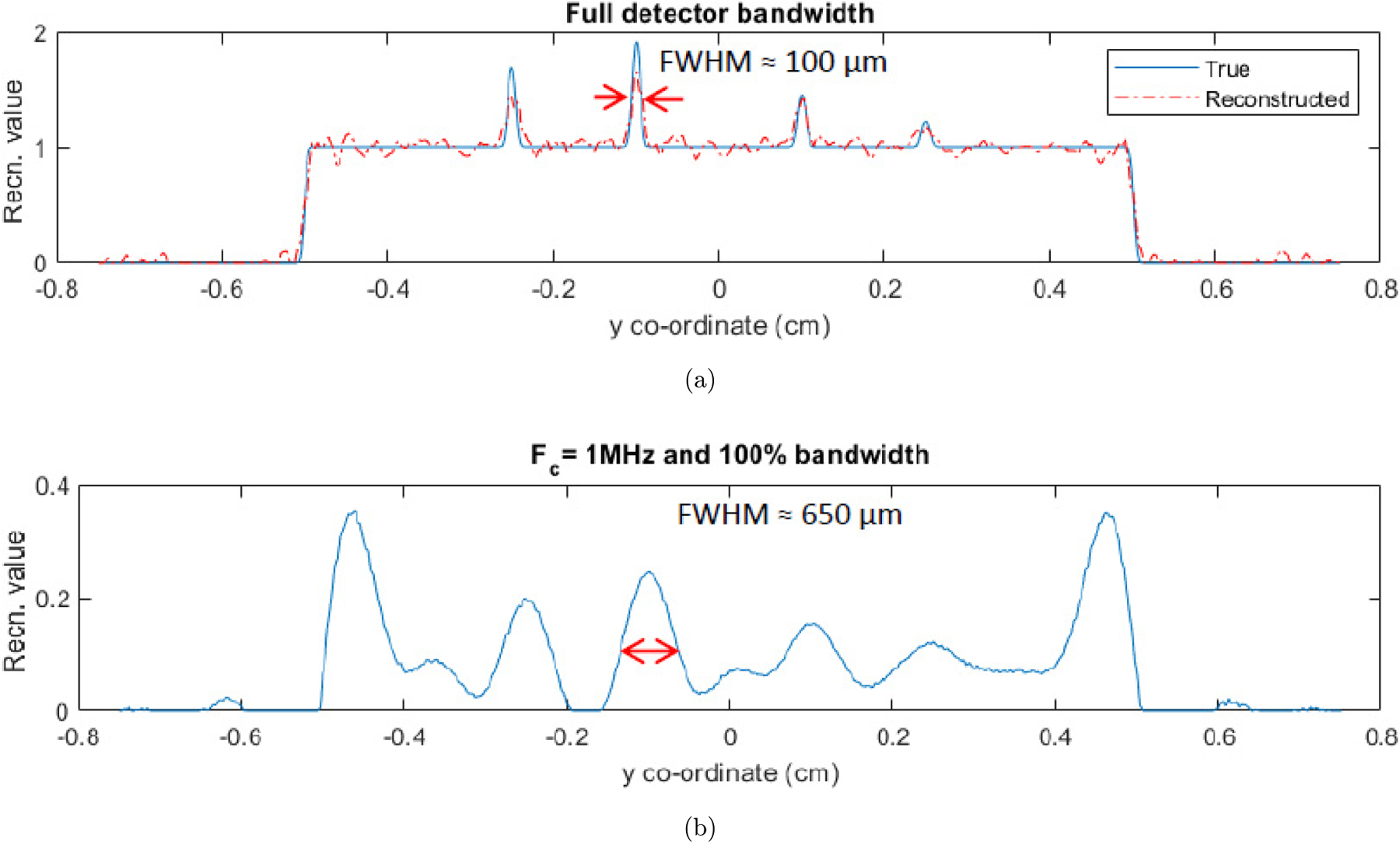

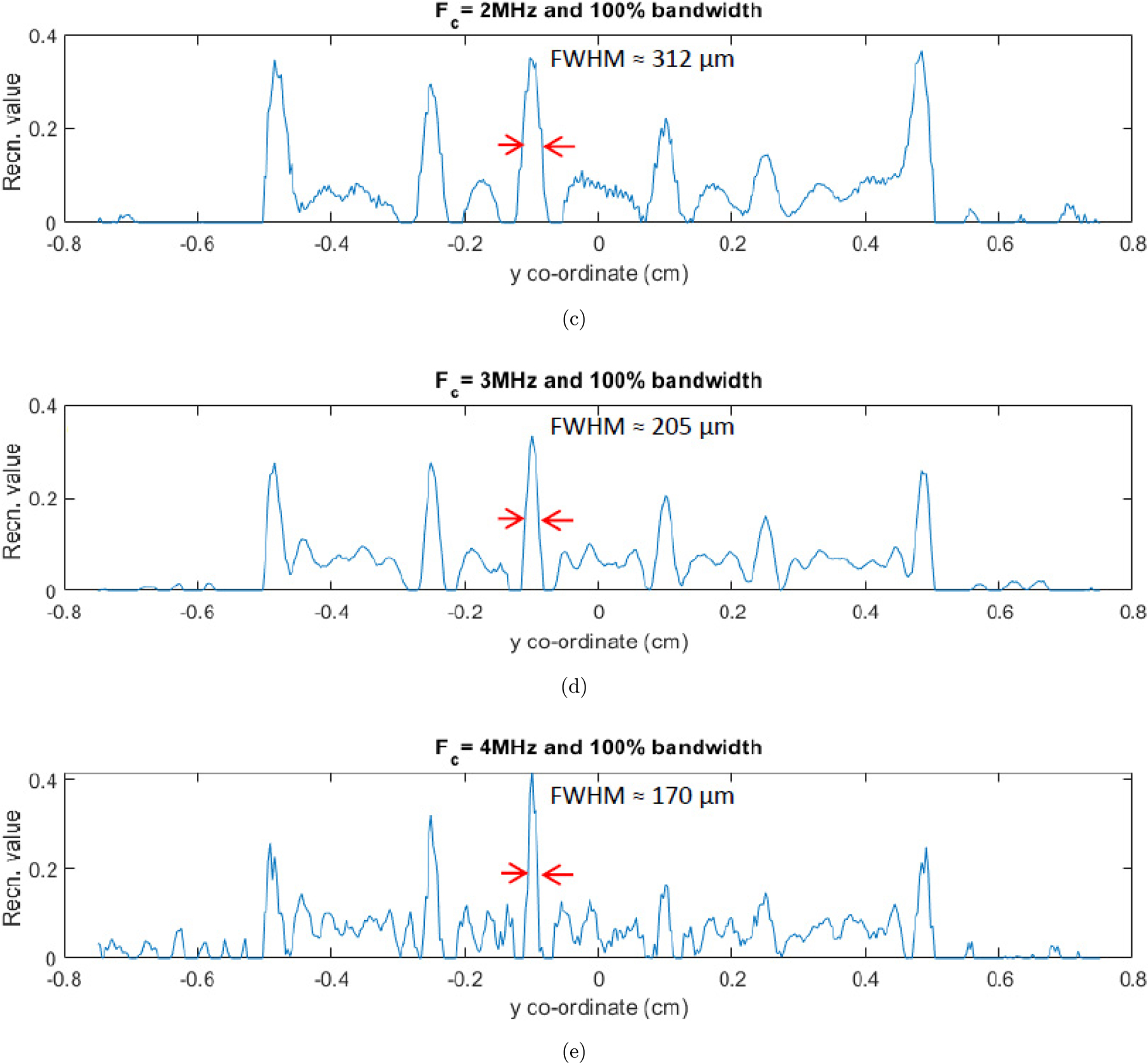
Numerical results: Profile plots of the true phantom and the full-view MF-LSQR reconstructions from numerical XACT data. Profile of (a) the true phantom and reconstructions with full frequency bandwidth, (b) $F_{c}=1\;\text{MHz}$ and 100% bandwidth, (c) $F_{c}=2\;\text{MHz}$ and 100% bandwidth, (d) $F_{c}=3\;\text{MHz}$ and 100% bandwidth, and (e) $F_{c}=4\;\text{MHz}$ and 100% bandwidth data.

We performed additional numerical studies to evaluate the contrast and resolution capabilities of the XACT. Four thin $\left(100\;µ\text{m}\right)$ lines with contrast between 1.2 and 1.9 with respect to the background as shown in Fig. 4(a). The forward measurements were computed at 1/64 mm resolution using about 10,000 quadrature points at 20 MHz sampling frequency. The resolution and quadrature points were chosen to be 1/32 mm and 5000, respectively, for reconstructions. The detection geometry was kept the same as the studies in Sec. 3.1. White Gaussian noise was then added to the XACT measurements to get data with 5 dB SNR. Model-based LSQR reconstruction was first performed using the data assuming full frequency bandwidth detection and depicted in Fig. 4(b). The corresponding profile plot is shown in Fig. 5(a) which compares reasonably well with the true phantom profile. Typically, the detection systems are bandlimited and the transducer's detection bandwidth is the main factor that characterizes the resolution of an imaging system. To study the resolution capability of XACT, the measurements were filtered with a Gaussian filter which mimics the frequency response of the detection system that is characterized by a central frequency $\left(F_{c}\right)$ and detection bandwidth. In this study, we used $F_{c}=1\text{,}\;2\text{,}\;3\;\text{and}\;4\;\text{MHz}$ with 100% bandwidth and corresponding LSQR reconstructions are demonstrated in Figs. 4(c)–4(f), respectively. To evaluate the resolution of these images, corresponding profiles are plotted in Figs. 5(b)–5(e) and the FWHM has been measured and tabulated in Table 2 along with the theoretical spatial resolution which for an acoustic detection system is given by $d={\text{λ}}_{\text{max}}\text{/}2$, with ${\text{λ}}_{\text{max}}$ being the wavelength corresponding to the highest frequency in the detection bandwidth.^4^ The relatively poor spatial resolutions (w.r.t. the theoretical resolution) for the reconstructions can be attributed to the smoothening effect of the regularization that is used to reduce the noisy artifacts in the reconstructions. For the testcases considered here, we see that the target with contrast (w.r.t. the background) as low as 1.2 was reasonably reconstructed. The contrast in XACT imaging comes from the difference in the X-ray absorption characteristics which in turn is associated with the densities of the materials. Moreover, signal SNR also plays a role in deciding the contrast reconstruction capability of the imaging system. For low SNR data, the noisy artifacts in the reconstructions can overshadow the structures with low contrast.

## 4. Ring Artifacts Correction for Experimental XACT Data

**Fig. 6.**
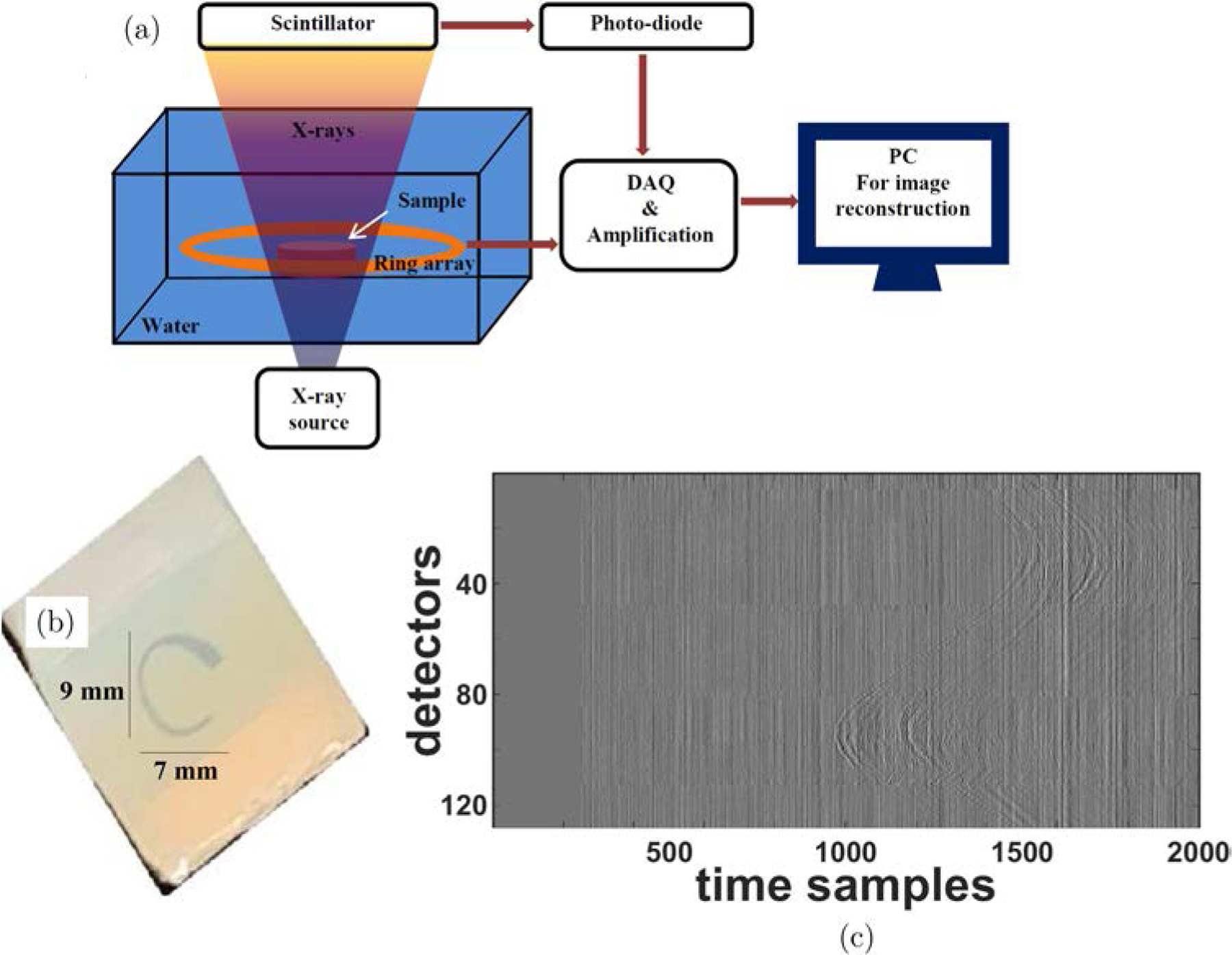
(a) Schematic of the XACT experimental setup, (b) image of the gelatin-based phantom with C-shaped lead target and (c) the sinogram of the XA measurements.

The efficacy of the proposed model-based ring artifacts corrected reconstruction algorithms is further studied on experimental XACT data. The schematic of the XACT experimental setup along with the photograph of the phantom and the XA sinogram carrying stripe pattern caused by the nonuniform detector response are displayed in Fig. 6.

**Fig. 7.**
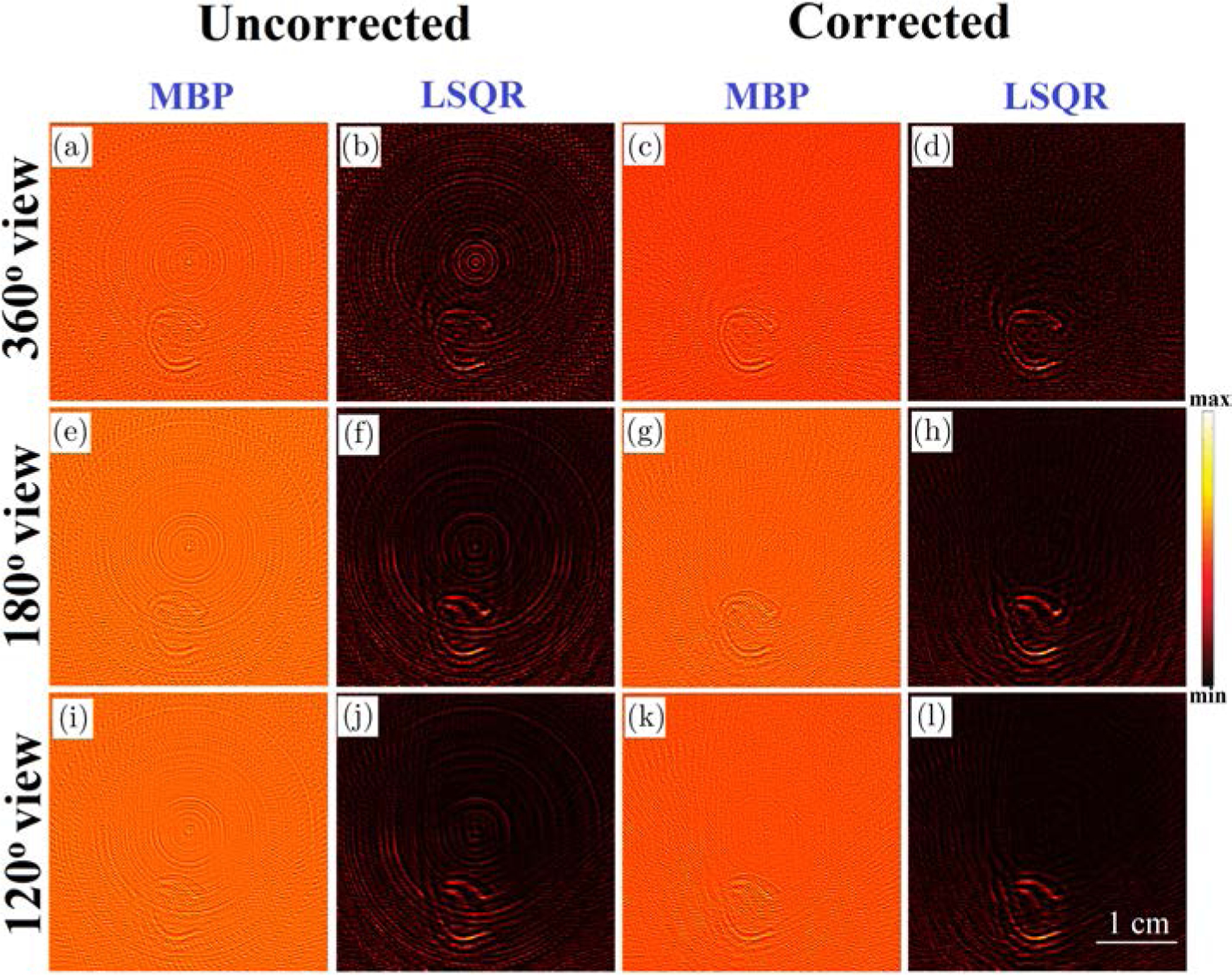
XACT experimental results: Conventional (a), (e), (i) MBP and (b), (f), (j) MF-LSQR reconstructions and ring-artifacts corrected (c), (g), (k) MBP and (d), (h), (l) MF-LSQR reconstructions for 360°, 180° and 120° view XA measurements.

**Fig. 8.**
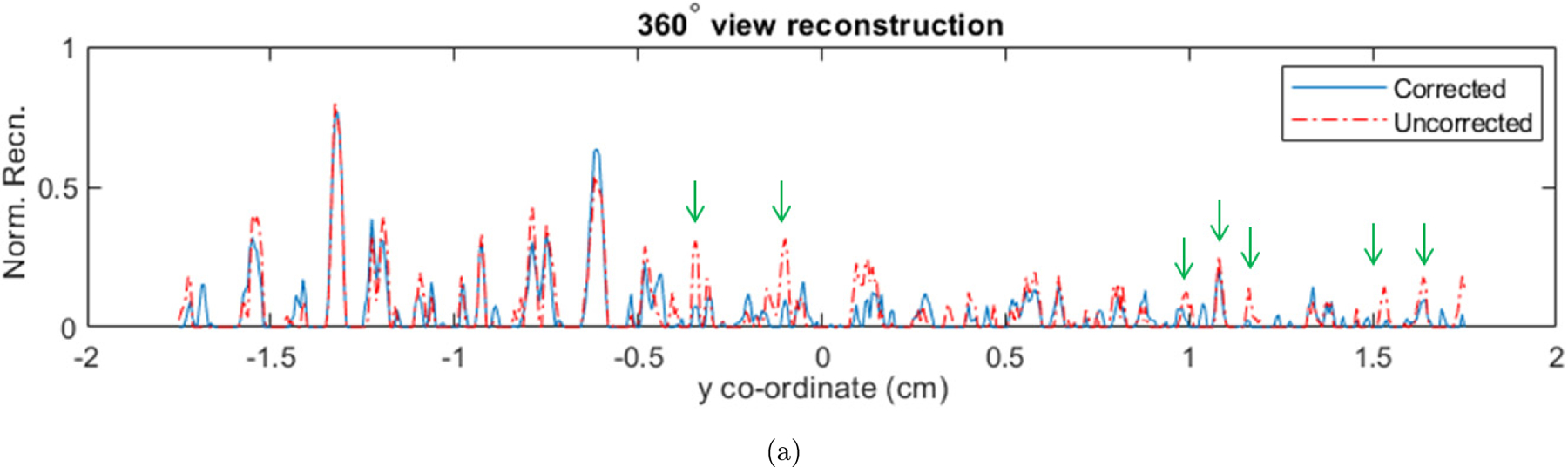

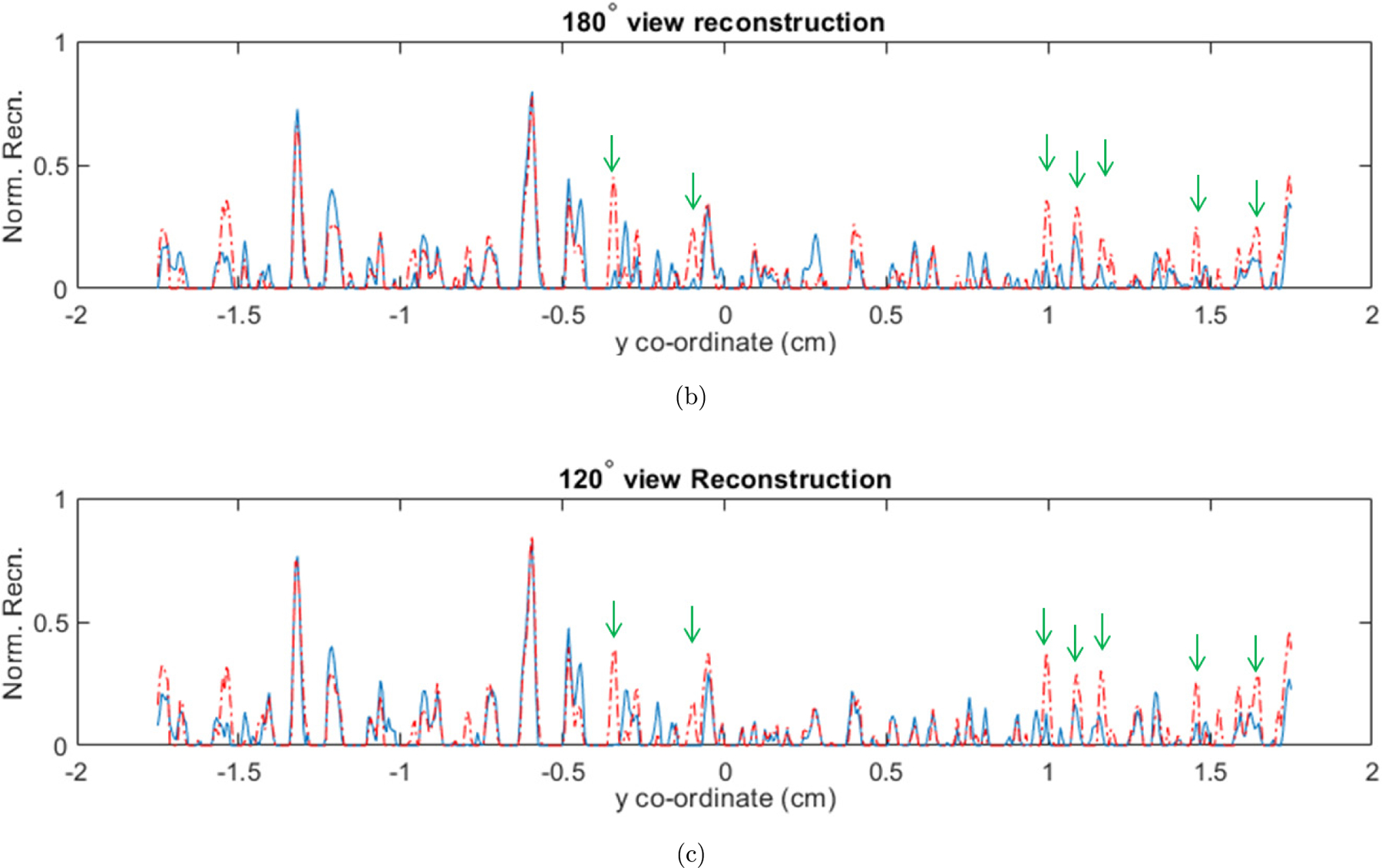
Profiles (along the central vertical line) of the MF-LSQR reconstructions from experimental XACT data with (a) 360°, 180° and 120° view measurements. Arrows indicate the effect of the ring artifacts on the profile.

A target (thin slice) in “C” shape, made of lead was fixed at the center of a gelatin phantom. The phantom and the ring shaped ultrasound detection array were placed in the water tank. Short X-ray pulses (pulse repetition rate of 10 Hz, and pulse width of 50 ns) were incident on the phantom. The XA waves caused by the thermoelastic expansion of the phantom were detected by each transducer element of the ring array and sent to the three-stage amplification and data acquisition system. The generated XA waves were sensed by a 128-element ultrasound ring-array (radius: 5 cm, PA probe, Doppler Co., Limited, Guangzhou, China) with 5 MHz central frequency and ≥ 60% bandwidth. To improve the SNR, the XA signals were averaged over 1500 pulses. Before performing reconstructions, the sinogram has been padded with zeros to account for the electromagnetic delay and the headwave.^4^ Conventional as well as the ring artifacts corrected model-based (MF-LSQR and MBP) XACT reconstructions were evaluated and displayed in Fig. 7. Corresponding contrast-to-noise ratios (CNRs) are provided in Table 3. The nonuniform detector response causes strong ring artifacts in the conventional model-based reconstructions Figs. 7(a), 7(b), 7(e), 7(f), 7(i) and 7(j). As expected, the proposed model-based algorithms are able to ameliorate the ring artifacts without any apparent loss in the contrast of the target Figs. 7(c), 7(d), 7(g), 7(h), 7(k) and 7(l). The profile plots for the uncorrected and corrected MF-LSQR reconstructions for the full and partial view measurements are provided in Fig. 8. The peaks in the profiles of the uncorrected reconstruction correspond to the ring artifacts in the images. This is also reflected in the CNR table (Table 3), where the ring artifacts corrected algorithm yields relatively better CNRs than its conventional counterparts. The proposed algorithm is equally effective in the full as well as limited view detection settings.

The nonuniform response of the multichannel DAQ yields vertical lines in the sinogram (Fig. 1(c)), which further produces ring artifacts in the XACT reconstructions. However, unlike the simulation (Fig. 1(c)), vertical lines in the sinogram obtained from the XACT experiment are not uniformly straight. From a closer observation, one can notice that these lines are made of piecewise uniform line segments (now shown in Fig. 4(c)). This is associated with the bundling of the cables from the transducers to the DAQ. Each set of cables bundled together will have a distinct nonuniform response thus resulting in distinct piecewise line segments in the sinogram. The reduced quality of the artifact-suppression for experimental XACT as compared to the simulations can indeed be attributed to this. Moreover, the attenuation and distortion of XA waves due to the finite-shaped lead target are also responsible for the noisy artifacts in the experimental XACT images. Other possible sources of error include the acoustic reflections due to acoustic mismatch between the target and the background, out-of-plane contribution to the XA signal, as well as inaccuracy in the radius and shape of the ring-array. Studies to correct these issues are ongoing.

## 5. Conclusion

In multichannel XA detection systems, nonuniform detector response is a problem that originates due to the interference between DAQ electronics and amplifier systems. This causes all sensor elements to have a simultaneous instantaneous gain in the acquired XA signal resulting in vertical lines (stripe pattern) in the XA sinogram and artifacts in the reconstructed images; ring artifacts in circular detection geometry. XACT systems yield low SNRs due to weak contrast, longer pulse widths, and lower pulse energy. Because of this, the ring artifacts in XACT are much stronger than other sister modalities such as PAT and TAT. However, this causes ring artifacts to appear more severe in XACT reconstruction, which is why removal of the artifacts in XACT is crucial for its clinical translation as a tool for tomographic imaging as well as radiation dosimetry. We proposed a correction method integrated into the model-based LS minimization approach to produce ring artifacts corrected reconstructions from corrupt, noisy XA measurements. Since the correction technique is fused with the model-based reconstruction algorithms, there are no additional computational costs associated. The proposed algorithm was tested on the numerical as well as experimental XACT datasets and produced desired results in the full as well as limited view detection geometries. Although the presented model-based correction algorithm does remove the ring artifacts while preserving the structures of the targets, the contrast among the reconstructed targets has some inaccuracy. Further studies will be performed to rectify this and the limited view problem to improve XACT and aid in its clinical translation.

## Figures and Tables

**Table 1.** Correlation coefficients for the corrected and uncorrected model-based reconstructions.

View	Algo	MB corrected	BW corrected	No corrected
360°	MF-LSQR	0.95	0.79	0.90
	MBP	0.44	0.45	0.21
180°	MF-LSQR	0.9	0.65	0.81
	MBP	0.34	0.31	0.17
120°	MF-LSQR	0.78	0.52	0.69
	MBP	0.29	0.25	0.12

**Table 2.** Theoretical and evaluated resolutions for MF-LSQR reconstructions.

$F_{c}\left(\text{MHz}\right)$	Theoretical resolution (µm)	Evaluated resolution (µm)
1	500	650
2	250	312
3	167	204
4	125	170

**Table 3.** Contrast to noise ratios for the corrected and uncorrected model-based reconstructions.

View	Algo	MB corrected	No corrected
360°	MF-LSQR	0.31	0.26
	MBP	0.0028	0.0029
180°	MF-LSQR	0.28	0.22
	MBP	0.0023	0.0022
120°	MF-LSQR	0.27	0.21
	MBP	0.0014	0.0010
